# Conformational Selectivity of Merocyanine on Nanostructured Silver Films: Surface Enhanced Resonance Raman Scattering (SERRS) and Density Functional Theoretical (DFT) Study

**DOI:** 10.3389/fchem.2022.902585

**Published:** 2022-06-13

**Authors:** Abhishek Das, Ridhima Chadha, Amaresh Mishra, Nandita Maiti

**Affiliations:** ^1^ Radiation & Photochemistry Division, Bhabha Atomic Research Centre, Mumbai, India; ^2^ Department of Chemistry, Sambalpur University, Sambalpur, Orissa; ^3^ Homi Bhabha National Institute, Mumbai, India

**Keywords:** merocyanine, surface-enhanced resonance Raman scattering (SERRS), density functional theory (DFT, ), structural and vibrational analysis, conformational surface selectivity

## Abstract

In this study, detailed structural and vibrational analysis of merocyanine has been investigated using Raman, surface enhanced Raman scattering (SERS) and surface-enhanced resonance Raman scattering (SERRS). The Raman, SERS and SERRS studies aided by density functional theoretical (DFT) calculations clearly established the prevalence of the trans- and cis-conformers of the protonated form of merocyanine (MCH^+^) in solid and acetonitrile solution. The binding characteristics of merocyanine adsorbed on nanostructured silver-coated films (SCFs) were investigated using excitation-dependent SERS, concentration-dependent SERRS and DFT studies. The conformers of merocyanine involved in the surface adsorption processes were recognized. The prominent marker bands observed at 1538 (ethylenic C=C stretch) and 1133 cm^−1^ (pyridinium C-N stretch) in the Raman spectrum of merocyanine in acetonitrile shifted to 1540 and 1126 cm^−1^, respectively on the nanostructured SCFs. The shift in the marker bands is associated with either the preferential binding of selective conformer or change in resonance equilibrium between the benzenoid and quinoid forms. The excitation wavelength dependent SERS spectrum infers that in addition to the major contribution from the electromagnetic enhancement, chemical (resonance) effect leads to the amplification of the 1540 cm^−1^ band. The concentration-dependent SERRS study showed maximum enhancement for the nanostructured SCFs functionalized with 1 μM concentration of merocyanine, indicative of monolayer coverage. For lower concentrations of merocyanine, the SERRS signal intensity reduced without any alteration in the peak positions. The SERRS study thus, revealed sub-nanomolar (0.1 nM) sensing of merocyanine using nanostructured SCFs with the analytical enhancement factor (AEF) of ∼ 10^10^ for the 1126 cm^−1^ and 1540 cm^−1^ Raman bands for MC concentration of 0.1 nM. In this study, combination of SERRS and DFT have clearly established the predominance of trans-MCH^+^ on the nanostructured silver surface with minor contribution from cis-MCH^+^, which remain exclusively bound to the surface *via* the phenoxyl ring O atom. This conformational surface selectivity of geometrical isomers of merocyanine using nanostructured surfaces can be further explored for energy efficient and economical separation of geometrical isomers.

## Introduction

Merocyanine (MC) dyes and their derivatives comprise an important class of heterocyclic compounds that consist of electron accepting and electron donating groups at the two terminals. These compounds exhibit remarkable solvatochromic behaviour (Murugan et al., 2011; Manzoni et al., 2016) displaying solvent-dependent hyperpolarizabilty (Levine et al., 1978; Reish et al., 2012; Siqueira et al., 2022) and have been used extensively in non-linear optics (Würthner et al., 2002). These dyes also find applicability in the field of medicine for diagnosis as non-invasive probes and as photosensitizers in photodynamic therapy (Allison et al., 2004; Bilici et al., 2021). The solvatochromic behavior of MC results in solvent-dependent shifts of the absorption maximum, which can be correlated to the variation between the two resonance structures, viz., the benzenoid (polar) and the quinoid (nonpolar) forms. The benzenoid form is predominant either in polar solvents or in solvents with high hydrogen bonding ability as well as large dielectric constant and the quinoid form is dominant in non polar solvents. NMR studies have shown that the protonated form (MCH^+^) undergoes photo induced *trans*-*cis* isomerization with ultraviolet light irradiation and remains in equilibrium between the *trans-* and *cis*-isomers (Steiner et al., 1978). The deprotonated form (MC) remains in the more stable *trans*-conformation in water and does not undergo photochemical and thermal isomerization to the *cis*-isomer. Deprotonation of the photochemically produced *cis*-MCH^+^ can be readily converted to *trans*-MC either via photochemical or thermal process. The structural changes in the molecule following photo-excitation are often investigated using the resonance Raman scattering (RRS) technique (Biswas and Umapathy, 1998, Biswas and Umapathy, 2001; Biswas et al., 2002; Nikolenko et al., 2019; Carvalho and Pimenta, 2020). The major drawback of RRS is the huge fluorescence background from the molecule or associated impurities that often mask the Raman signal. The fluorescence background in RRS is usually overcome with SERS (Surface enhanced Raman scattering). SERS is a highly sensitive spectroscopic technique that in addition to the quenching of the fluorescence background leads to multifold intensity enhancement of the Raman bands from analytes adsorbed on the metal nanoparticles (NPs) surface (Thomas et al., 2005; Biswas et al., 2009; Thomas et al., 2010; Saviello et al., 2019; Yang et al., 2019; Langer et al., 2020; Das et al., 2021; Dhayagude et al., 2021). The intensity enhancement in SERS usually originates from two cooperative mechanisms, the “long-range” electromagnetic (EM) (Wu et al., 2008) and the “short-range” chemical (Campion et al., 1995) effect.

Silver (Ag) and gold (Au) nanoparticles (NPs) show huge SERS enhancement and are ideal candidates due to their encouraging physicochemical properties. The surface plasmon resonance (SPR) band of these noble metal NPs appear in the visible region and they display large scattering cross sections making them suitable for molecular labeling studies (Jain et al., 2007; Jacob et al., 2011; Rycenga et al., 2011; Ringe et al., 2013; Chadha et al., 2021b; Das and Maiti, 2022). SERS gains high sensitivity from plasmon-enhanced excitation and scattering, thereby allowing for rapid, non-invasive *in situ* detection of target molecules. Recently, many review articles have focused on the advantages, reliability and future developments of SERS (Medříková et al., 2019; Fan et al., 2020; Langer et al., 2020; Pérez-Jiménez et al., 2020; Li et al., 2021; Han et al., 2022). Applications of the SERS technique in biochemical and medical analysis are being discussed (Chaloupková et al., 2018; Ranc et al., 2018; Zong et al., 2018; Szaniawska and Kudelski, 2021). Lately, SERS is being used for studying in-situ surface-catalyzed chemical oxidation (Huang et al., 2010; Kang et al., 2013; Chadha et al., 2014; Kumar et al., 2019a) as well as charge rearrangement reactions (Dhayagude et al., 2016; Das et al., 2019). Traces of drugs (Edwin et al., 2017; Kumar et al., 2019b), toxic heavy metal ions (Wang et al., 2016; Guselnikova et al., 2017; Chadha et al., 2021a), insecticides (Dissanayake et al., 2019; Mane et al., 2020; Chadha et al., 2022), etc. can be detected using SERS, which also provides valuable structural and vibrational information pertaining to the metal-analyte interaction (Thomas et al., 2013; SenGupta et al., 2014; Maiti et al., 2015, Maiti et al., 2016; Das et al., 2019; Mirajkar et al., 2020). The advantages of RRS and SERS can be combined in surface-enhanced resonance Raman scattering (SERRS) (Biswas et al., 2006; Biswas et al., 2008; Kitahama and Ozaki, 2016; Nicolson et al., 2018; Litti et al., 2020) technique that displays very high sensitivity and selectivity.

**SCHEME 1:**
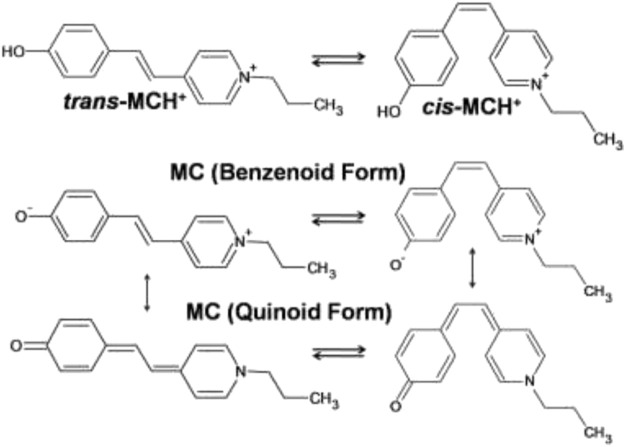
Molecular structures of the *trans*- and *cis*-conformers of protonated (MCH^+^) and deprotonated (MC) forms of merocyanine dye [4'-(hydroxystyryl)-4-propylpyridinium bromide].

In this study, the SERRS technique has been exploited for studying the binding characteristics of MC [4'-(hydroxystyryl)-4-propylpyridinium bromide] adsorbed on nanostructured silver-coated films (SCFs). The protonated form (MCH^+^) contains a positively charged pyridinium ring and a neutral phenoxyl ring connecting the ethylenic C=C bond that remains in equilibrium between the *trans*- and *cis*-conformers. The molecular structure of the *trans*- and *cis*-conformers of MCH^+^ is shown in Scheme 1. The main objective of this study is to identify the most prevalent conformer of MCH^+^ in solid as well as solution and to recognize the species that is adsorbed on the nanostructured SCFs. In solution as well as on the nanostructured film surface, MCH^+^ may undergo deprotonation and remain as MC. The deprotonated form may also undergo *trans*-*cis* isomerization and exist either as benzenoid or quinoid as is shown in Scheme 1. In order to identify the dominance of the *trans*-/*cis*-conformer of MCH^+^/MC in solid, solution, nanostructured surface and to understand the binding characteristics; Raman, SERRS and DFT studies were performed. The binding characteristics were probed by monitoring the changes in the Raman spectral features measured on the nanostructured SCFs with respect to that in solid and solution. To the best of our knowledge, this is the first comprehensive report discussing the structural and vibrational features of MCH^+^/MC adsorbed on nanostructured silver surface and identifying the predominant conformer on the surface. The experimental results are supported with DFT calculations. Earlier reports on the infrared reflection-absorption (Itoh et al., 1992), resonance Raman intensity analysis (Leng et al., 2004) and SERRS (Tsukada et al., 1989; Mineo and Itoh, 1991; Pajchrowski et al., 2007) studies of MC have indicated the presence of *trans*-conformation on the metal surface, which is in agreement with this study. Thus, multi-faceted application of MCH^+^/MC in bio sciences, materials and chemistry has motivated the present study, with an aim to gain thorough understanding of its molecular level behavior on the nanostructured surface of SCFs.

## Materials and Methods

### Chemicals

Silver nitrate (AgNO_3_), formamide, acetonitrile, n-propylbromide, γ-picoline, 4-hydroxybenzaldehyde and ethanol that were used for the synthesis of 4'-(hydroxystyryl)-4-propylpyridinium bromide (MCH^+^) and nanostructured silver-coated films (SCFs) were obtained from S. D. fine chemicals, India. All the solutions and SCFs were kept in the dark to avoid any photochemical reaction.

### Synthesis of 4'-(Hydroxystyryl)-4-Propylpyridinium Bromide (MCH^+^)

**SCHEME 2:**
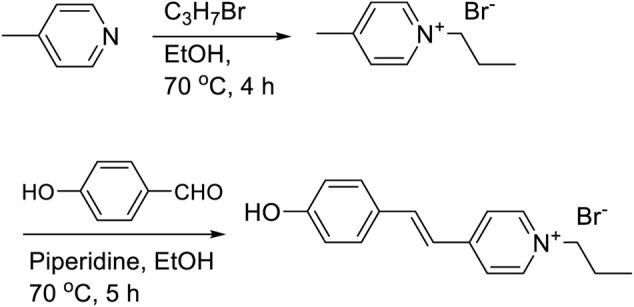
Synthetic protocol of merocyanine dye (MCH^+^).

4'-(hydroxystyryl)-4-propylpyridinium bromide (MCH^+^) was prepared by the reaction of *n*-propylbromide with *γ*-picoline followed by Knoevenagel condensation with 4-hydroxybenzaldehyde in ethanol. The dye was purified by recrystallization in ethanol. The synthetic protocol is shown in Scheme 2.

### Synthesis of Nanostructured Silver-Coated Film (SCFs)

Nanostructured silver-coated films (SCFs) were prepared by taking glass slides that were thoroughly cleaned with chromic acid, washed with Millipore water and then dried in oven. The clean glass slides were dipped in 5 × 10^–2^ mol L^−1^ solution of AgNO_3_ in formamide for two and half hours. Formamide reduces Ag^+^ ions to Ag^0^ and the nanoparticles (NPs) formed get deposited onto the glass slides resulting in the formation of stable nanostructured SCFs (Sarkar et al., 2005; Sarkar et al., 2009; Maiti et al., 2013). MCH^+^/MC functionalized SCFs were prepared by dipping the nanostructured SCFs in acetonitrile solutions of varying concentrations of MCH^+^/MC for 15 min. The functionalized SCFs were removed from the acetonitrile solution, washed thoroughly with Millipore water and then air-dried. The functionalized SCFs (MC-SCFs) were then characterized using Atomic Force Microscopy (AFM), UV-Visible absorption, SERS and SERRS.

### Instrumentation

The surface morphology of the SCFs and MC-SCFs was analyzed using AFM (Model: A-100 AFM instrument, A.P.E. Research, Italy). All images were measured in non-contact mode using aluminum coated n-type silicon cantilever (HQ:CSC17/Al BS, μMasch, Germany) with the force constant, 0.18 N/m and frequency, 13 kHz. The radius of uncoated tip was 8 nm with a height of 12–18 μm. The UV-Vis absorption spectra were recorded using a JASCO V-650 spectrophotometer. The Raman spectrum of solid MCH^+^ was recorded at room temperature (RT) by placing the powdered sample on a glass slide and collecting the scattered light at the 180° scattering geometry with a ×50 LWD (long working distance) objective using the 785 nm diode laser. For the Raman measurements of MCH^+^/MC in acetonitrile solution, the sample was taken in a standard 1 × 1 cm^2^ cuvette and the scattered light was collected at 180° scattering geometry and the signal detected using a charge-coupled device (LabRAM HR800, Horiba Jobin Yvon, France) together with an edge filter for 785 nm. The SERRS spectrum of MC-SCFs with varying MC concentrations was recorded at RT using the 514.5 nm excitation line, from the Ar ion laser. The SERS spectrum for MC concentration of 1 μM was measured at different excitation wavelengths with 632.8 (He-Ne) and 785 nm (diode) lasers. The spectrometer was calibrated using the Raman spectrum of silicon wafer at 520 cm^−1^. All the Raman, SERS, and SERRS spectra were recorded with 600 grooves/mm grating. The laser power at the sample surface was 0.5, 1, and 10 mW for the excitation wavelengths, 514.5, 632.8, and 785 nm and the spectral resolution was found to be 1.6, 1.1, and 0.7 cm^−1^, respectively. The diameter of the laser spot at the sample surface was 300, 200, and 500 μm, respectively for the 514.5, 632.8, and 785 nm excitation wavelengths.

## Computational Methods

In order to gain insight into the experimental Raman spectrum, the geometry of both the *trans*- and *cis*-conformers of MCH^+^ and MC were optimized using DFT [Gaussian 09 program (Frisch et al., 2009)] with B3LYP functional (Becke, 1993) and 6–31+G* as well as DGDZVP basis sets. At the optimized geometry of each conformer of MCH^+^ and MC, the molecular vibrations were computed and the theoretically calculated vibrations were compared with the experimentally observed Raman spectrum in solid and solution. The molecular structure of the *trans*- and *cis*-conformers of MCH^+^ and MC was also optimized by considering the effect of acetonitrle as the solvent. At the optimized geometries, the time-dependent density functional theory (TDDFT) calculation was performed and the computed absorption spectrum for the *trans*- and *cis*-conformers of protonated and deprotonated forms in acetonitrile was compared with the experimental absorption spectrum of merocyanine recorded in acetonitrile. Geometry optimization was also performed for the simplistic model, viz, Ag_4_ complexes of the *trans*- and *cis*-conformers of MCH^+^ and MC (*trans*-MCH^+^-Ag_4_, *cis*-MCH^+^-Ag_4_, *trans*-MC-Ag_4,_ and *cis*-MC-Ag_4_), where LANL2DZ basis set was used for Ag. The TDDFT calculations and vibrational frequencies were computed at the optimized geometries of the complexes. The absence of imaginary frequency ensured that the optimized molecular structures correspond to local minimum on the potential energy surface and not to saddle points. The computed vibrations at the optimized geometries of the *trans*- and *cis*-conformers of MCH^+^ and MC and their Ag_4_ complexes were then compared with the Raman spectrum of MCH^+^/MC in solid and solution and the SERS spectrum. The computed absorption spectrum of the Ag_4_ complexes of *trans*- and *cis*-conformers of MCH^+^ and MC was compared with the absorption spectrum of the merocyanine functionalized SCFs.

## Results and Discussion

### Computational Results

**FIGURE 1:**
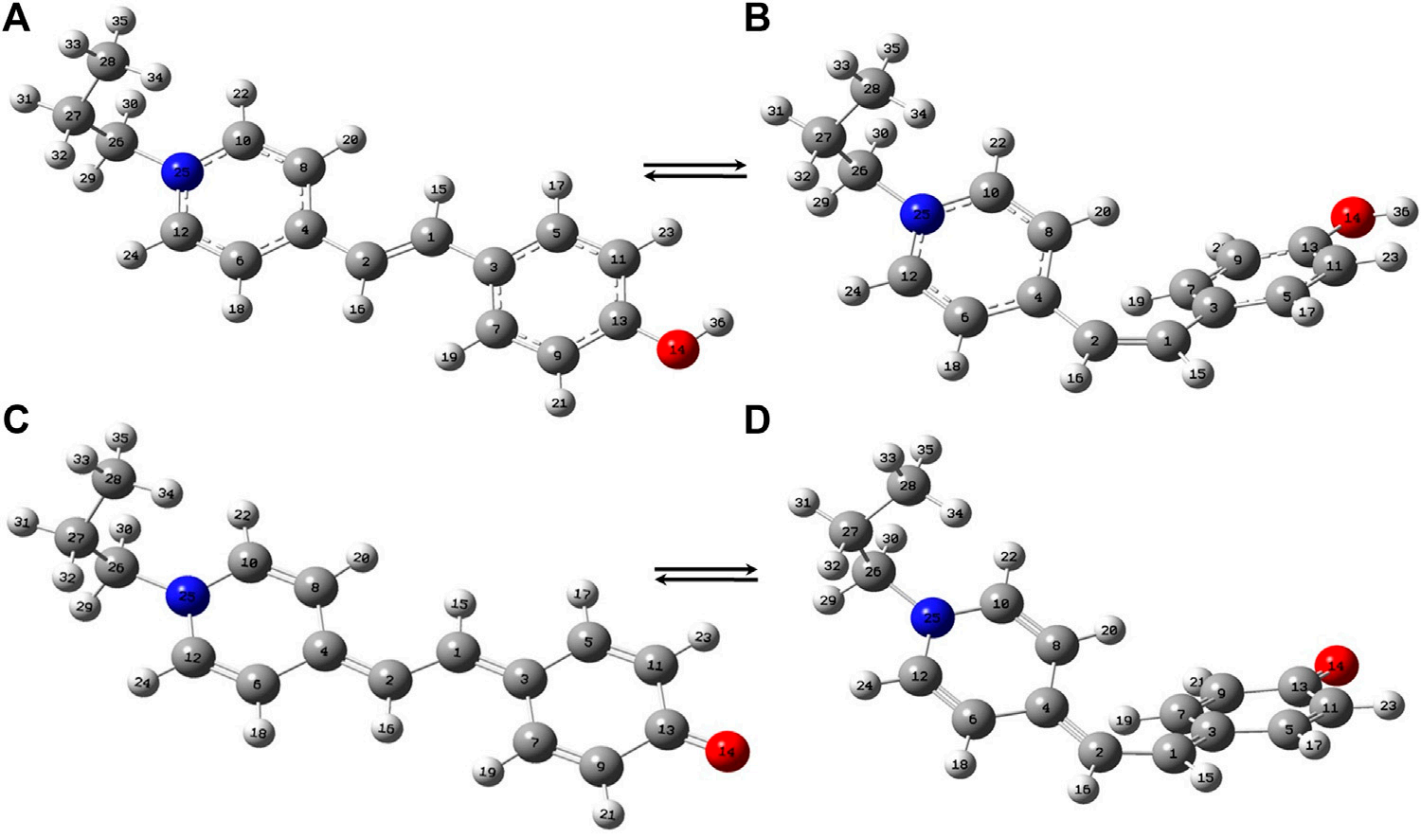
Optimized molecular structures of **(A)** *trans*-MCH^+^, **(B)** *cis*-MCH^+^, **(C)** *trans*-MC and **(D)** *cis*-MC. The color codes used to identify the atoms are O (red), N (blue), C (grey) and H (white).

The *trans*- and *cis*-conformers of MCH^+^ in their ground electronic state (S_0_) were optimized using DFT (B3LYP) method with 6–31+G* and DGDZVP basis sets. In order to know the relative stability of the *trans*- and *cis*-conformers of MCH^+^, their minimum energies at the optimized structure were compared. The results obtained from both 6–31+G* and DGDZVP basis sets, showed that *trans*-MCH^+^ is more stable than *cis*-MCH^+^ by an energy of 7.22 kcal mol^−1^ (0.31 eV). Since both the basis sets gave similar results, further calculations were carried out using the 6–31+G* basis set. The optimized structures of the *trans*-MCH^+^ and *cis*-MCH^+^ along with their atom numbering is shown in Figures 1A,B. The absence of imaginary vibrational frequency for *trans*-MCH^+^ and *cis*-MCH^+^ confirmed that the optimized geometries correspond to local minima on the potential energy surface. The protonated form (MCH^+^) may get deprotonated and remain as MC in solution and on the SCFs. Hence, geometry optimization was also carried out for *trans*-MC and *cis*-MC and their minimum energy computed. The computed energies of *trans*-MC and *cis*-MC at the B3LYP/6–31+G* level of theory indicated that the *trans*-conformer is more stable in comparison to the *cis*-conformer by an energy of 6.37 kcal mol^−1^ (0.27 eV). The optimized molecular structures of *trans*-MC and *cis*-MC are shown in Figures 1C,D, respectively. The vibrational frequencies for both *trans*-MC and *cis*-MC were computed at the optimized geometries. In order to identify the prevalence of *trans*-MCH^+^, *cis*-MCH^+^, *trans*-MC, and *cis*-MC in solid and acetonitrile solution, the computed Raman spectrum of each conformer was compared with the observed Raman spectrum in solid and solution. The computed “Raman intensity” in each case refers to the “Raman activity” (Neugebauer et al., 2002) as implemented in Gaussian 09. For a one-to-one correspondence of the computed Raman activity with the experimental Raman spectrum, the calculated frequencies were scaled down by a factor of 0.95 and the Raman bands were broadened with a Lorentzian function of 10 cm^−1^ full width at half maximum (FWHM). As discussed later, it is observed that the scaled vibrations show reasonable agreement with the experimental Raman spectrum of MCH^+^ in solid and acetonitrile solution. The absorption spectrum computed at the optimized geometries of *trans*-MCH^+^, *cis*-MCH^+^, *trans*-MC and *cis*-MC conformers in acetonitrile using TDDFT method was compared with the experimental absorption spectrum of merocyanine in acetonitrile and the observations are discussed later.

In order to identify the predominance of *trans*-MCH^+^, *cis*-MCH^+^, *trans*-MC, and *cis*-MC on the surface of SCFs, the simplistic model with Ag_4_ complexes for all forms; viz., *trans*-MCH^+^-Ag_4_, *cis*-MCH^+^-Ag_4_, *trans*-MC-Ag_4_, and *cis*-MC-Ag_4_ were optimized using B3LYP functional with 6–31+G* basis set and LANL2DZ basis set for Ag. The absence of imaginary vibrations at the optimized geometries confirmed that *trans*-MCH^+^-Ag_4_, *cis*-MCH^+^-Ag_4_, *trans*-MC-Ag_4_, and *cis*-MC-Ag_4_ correspond to local minimum on the potential energy surface and not to saddle points. The computed Raman spectrum of these complexes at different excitation wavelengths was compared with the SERS and SERRS spectrum measured at 632.8 and 514.5 nm. The absorption spectrum obtained from TDDFT calculation for *trans*-MCH^+^-Ag_4_, *cis*-MCH^+^-Ag_4_, *trans*-MC-Ag_4_ and *cis*-MC-Ag_4_ complexes was compared with the absorption spectrum of merocynaine functionalized SCFs. The optimized parameters, viz., the bond distances, bond angles and the dihedral angles connecting the ethylenic C=C bond are displayed in Supplementary Table S1. The optimized bond distances clearly suggest that while *trans*-MCH^+^, *cis*-MCH^+^ and their Ag_4_ complexes remain in the benzenoid form, *trans*-MC, *cis*-MC and their Ag_4_ complexes exist in the quinoidal form. The comparison of the computed Raman spectrum under preresonance excitation with the SERRS spectrum as discussed later clearly indicates the predominance of the *trans*- or *cis*-conformer of the protonated species of the analyte on the surface of SCFs.

### UV-Visible Absorption Study

**FIGURE 2:**
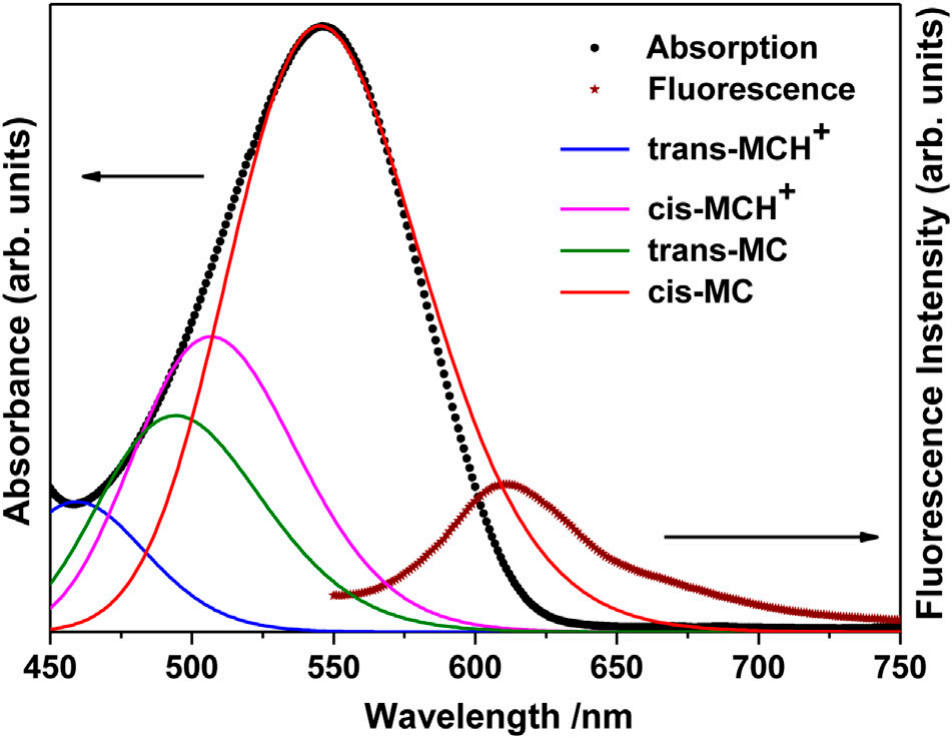
Absorption (Concentration: 10 μM) and fluorescence (Concentration: 0.1 μM) spectrum of merocyanine in acetonitrile solution. The computed absorption spectrum of *trans*-MCH^+^, *cis*-MCH^+^, *trans*-MC, and *cis*-MC in acetonitrile solvent is scaled and red-shifted by 32 nm to have a realistic resemblance with the experimental spectrum.

The UV-visible absorption spectrum of MCH^+^/MC in acetonitrile solution (10 μM) is shown in Figure 2. The absorption band in the visible region (450–750 nm) is broad and structureless and is attributed to the intramolecular charge transfer (ICT) transition from the electron rich phenoxyl group to the electron deficient pyridinium moiety. It is observed from Figure 2 that the absorption maximum appears at 546 nm with full width at half maxima (FWHM) of 85 nm. In the ground electronic state (S_0_), MC remains protonated as MCH^+^ and exists either in the *trans*- or *cis*-configuration around the ethylenic C=C bond. As observed from the computational results, the *trans*-MCH^+^ is energetically more stable than *cis*-MCH^+^. The positive charge in MCH^+^ is centered on the pyridinium moiety. It is known that in various solvents, MC exists in a resonance balance between the benzenoid and quinonoid forms (Tsukada et al., 1989) with the dominance of former in polar solvents and latter in non polar solvents. Moreover, in polar solvents, the benzenoid form dominates in S_0_ and the quinonoid form dominates in the excited state. The electronic excitation from the ground to the excited state, thus, involves change from the benzenoid to the quinonoid form, which is accompanied by significant changes in the ethylenic C=C bond and the pyridinium and phenoxyl rings connecting the ethylenic C=C bond. In order to confirm the prevalence of *trans*-MCH^+^, *cis*-MCH^+^, *trans*-MC, and *cis*-MC in acetonitrile solution, the computed absorption spectrum (TDDFT) for these conformers in acetonitrile is included in Figure 2. The computed absorption maximum for *trans*-MCH^+^ and *cis*-MCH^+^, was observed at 427.2 and 474.7 nm with the oscillator strength of 1.0257 and 0.3617, respectively. Similarly, the calculated absorption maximum and oscillator strengths for *trans*-MC (*cis*-MC) was found to be 458.4 (513.8) nm and 1.1726 (0.7285), respectively. In order to have a realistic resemblance of the experimental absorption spectrum with the computed spectrum for all conformers, the latter was scaled and red-shifted by 32 nm and broadened with a Gaussian function of 3000 cm^−1^ FWHM. The figure clearly shows the dominance of the cis-MC form in acetonitrile solution. The fluorescence spectrum of MC in acetonitrile recorded at 532 nm excitation is also included in Figure 2. It is observed from the figure that fluorescence maximum appears at 612 nm with FWHM of 57 nm.

**FIGURE 3:**
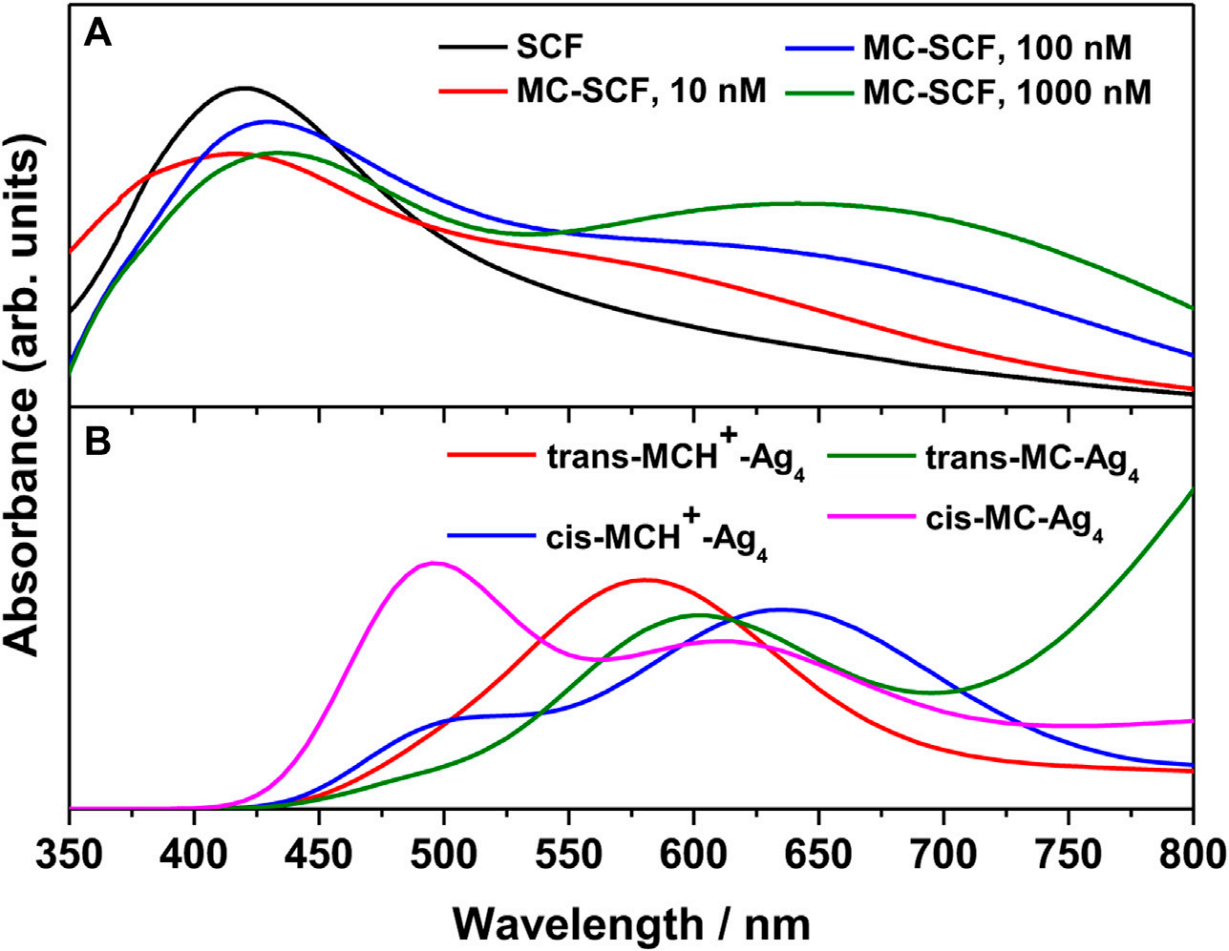
**(A)** Surface plasmon resonance (SPR) bands of bare nanostructured SCF and MC-SCF with varying concentrations of MC. **(B)** The computed absorption spectrum of *trans*-MCH^+^-Ag_4_, *cis*-MCH^+^-Ag_4_, *trans*-MC-Ag_4_ and *cis*-MC-Ag_4_ complex.

The UV-Vis absorption spectrum of the bare nanostructured SCF and MC-SCFs with varying concentrations of MCH^+^/MC was recorded. The absorption spectrum of the bare SCF is shown in Figure 3A. The figure shows the appearance of a broad band with maximum at 419.6 nm, attributed to the bulk-like surface plasmon resonance (BL-SPR) band (Le Ru and Etchegoin, 2012; Dutta Roy et al., 2018; Das et al., 2020; Chadha et al., 2021b; Chadha et al., 2022). It is known that the BL-SPR band of the metal NPs strongly depends on the shape, size and the extent of aggregation of the particles in addition to the dielectric constant of the medium as well as the surface adsorbed species (Jana et al., 2016). The absorption spectrum of the MC-SCFs with varying MC concentrations (10, 100, and 1000 nM) is also included in Figure 3A. It is observed from the figure that for MC-SCFs with 10 nM MC, the absorbance at 419.6 nm due to the BL-SPR band gradually reduces in intensity with slight blue shift to 416.2 nm along with the appearance of a shoulder around 500–750 nm. Upon further increasing the MC concentration to 100 and 1,000 nM; owing to the adsorption of the analyte on the surface of SCFs, the BL-SPR band red-shifts to 430 and 433 nm, respectively. For the MC-SCFs with 100 and 1,000 nM concentrations of MC, in addition to the red-shifting of the BL-SPR band, lower energy peaks were found to appear with maxima around 618 and 641 nm. These lower energy peaks were attributed to the surface-like surface plasmon resonance (SL-SPR) band (Le Ru and Etchegoin, 2012; Dutta Roy et al., 2018; Das et al., 2020; Chadha et al., 2021b; Chadha et al., 2022) that arises due to the dipole-dipole interactions of the higher aggregated particles. From the figure, it is observed that increase in MC concentrations resulted in the lowering of energy of the SL-SPR band with increased magnitude and concomitant broadening. The red-shifting and broadening of the SL-SPR band was attributed to the presence of higher aggregated particles and is dependent on the analyte concentration as well as the electronic transitions associated with the analyte (Chowdhury et al., 2003; Willets and Van Duyne, 2007; Pal et al., 2010). For a better understanding of the experimental results, the absorption spectrum of *trans*-MCH^+^-Ag_4_, *cis*-MCH^+^-Ag_4_, *trans*-MC-Ag_4_, and *cis*-MC-Ag_4_ was computed with TDDFT method and the results are included in Figure 3B. The absorption maximum for *trans*-MCH^+^-Ag_4_ and *cis*-MCH^+^-Ag_4,_ was observed at 571.4 and 663.5 nm along with the oscillator strengths of 0.0469 and 0.0361, respectively. Similarly, the computed absorption maximum for *trans*-MC-Ag_4_ and *cis*-MC-Ag_4_ was observed at 621.3 and 628.4 nm with the oscillator strengths of 0.0479 and 0.0369, respectively. For comparison of the experimental and calculated absorption spectrum, the computed spectrum of all conformers was scaled and broadened with a Gaussian function of 1500 cm^−1^ FWHM. The figure clearly shows that all the conformers, *trans*-MCH^+^-Ag_4_, *cis*-MCH^+^-Ag_4_, *trans*-MC-Ag_4_, and *cis*-MC-Ag_4_ have a broad absorption band with reasonable absorbance in the region from 550–700 nm.

### Atomic Force Microscopy Analysis

**FIGURE 4:**
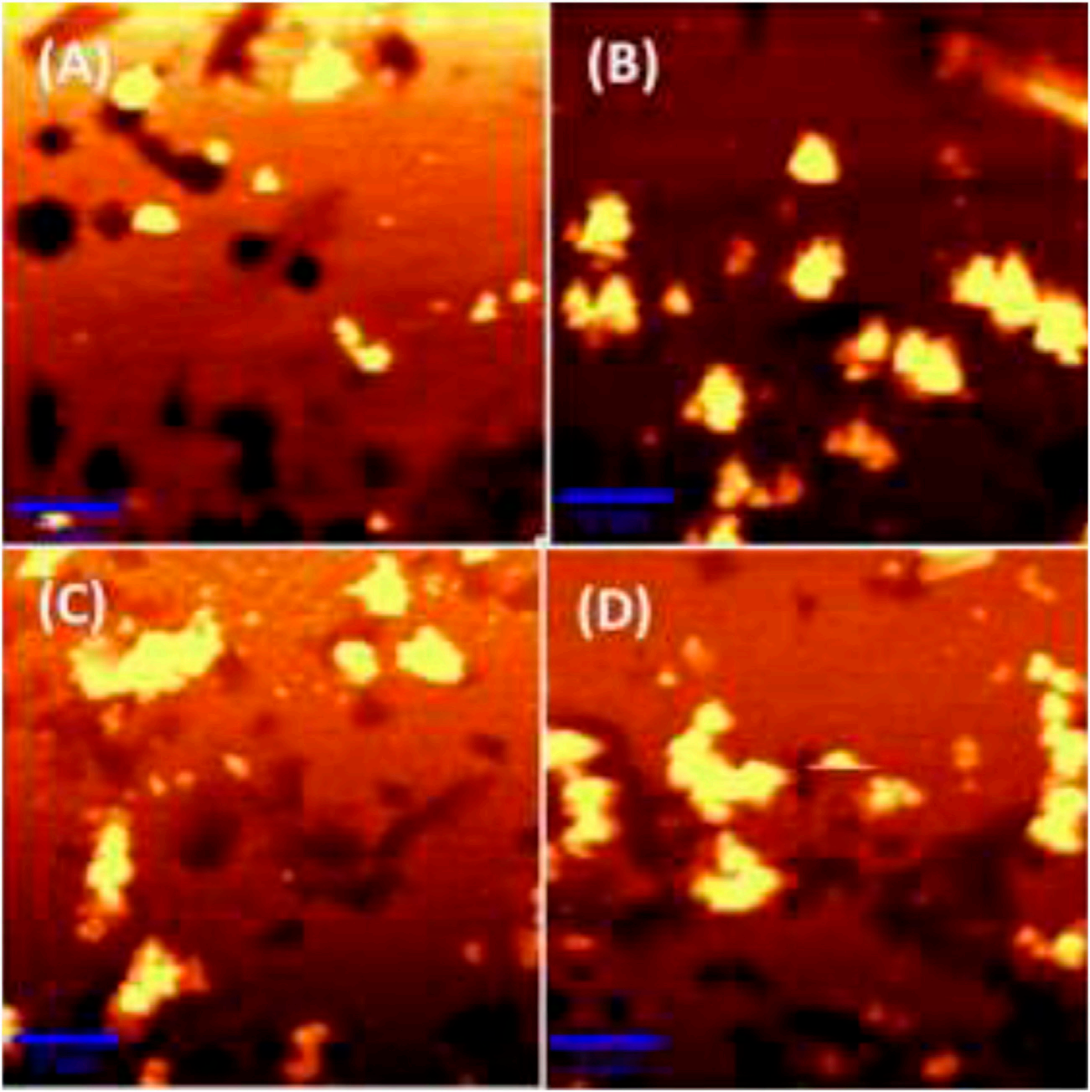
AFM images of **(A)** bare nanostructured SCF, **(B)** MC-SCFs (MC concentration, 10 nM), **(C)** MC-SCFs (MC concentration, 100 nM), and **(D)** MC-SCFs (MC concentration, 1000 nM).

Atomic Force Microscopy (AFM) images of bare nanostructured SCF and MC-SCFs with MC concentrations of 10, 100, and 1000 nM were recorded and are shown in Figure 4. The AFM image as displayed in Figure 4A shows the formation of polygonal shaped particles with an average size of 100 nm. The AFM image of MC-SCF with MC concentration of 10 nM clearly indicates the presence of aggregated particles with an average size of 250 nm as shown in Figure 4B. The AFM images of MC-SCFs with 100 nM and 1000 nM concentrations of MC are included in Figures 4C,D. From the AFM images (Figures 4C,D) the presence of aggregated particles with the particles approaching each other forming chain like structures is evident.

### Raman Spectra of Merocyanine and its Vibrational Assignments

**FIGURE 5:**
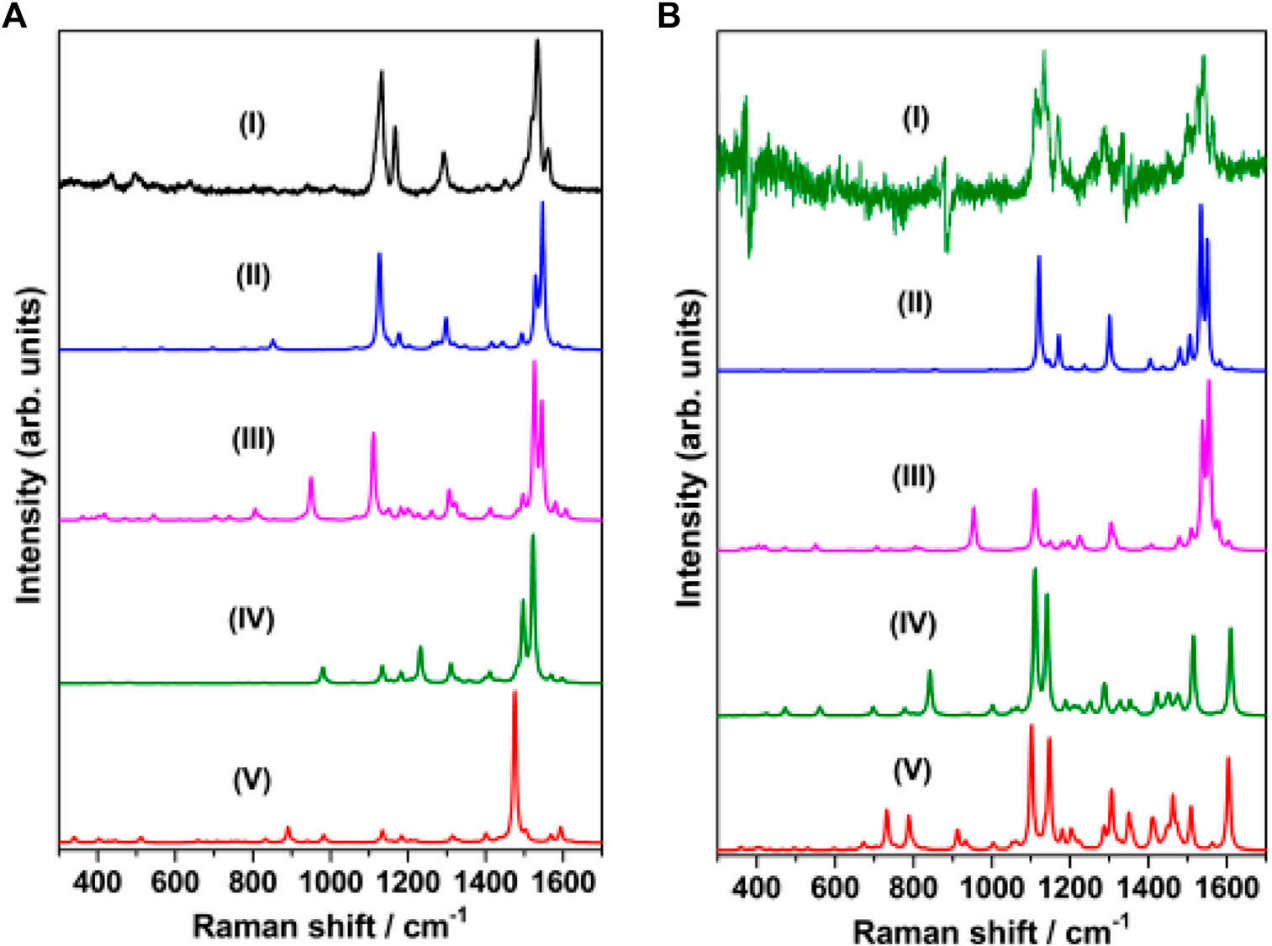
**(A)** Raman spectrum of merocyanine (I) solid, recorded at 785 nm excitation. B3LYP/6–31+G* computed Raman spectrum of (II) *trans*-MCH^+^, (III) *cis*-MCH^+^, (IV) *trans*-MC and (V) *cis*-MC. **(B)** Raman spectrum of merocyanine (I) in acetonitrile, recorded at 785 nm excitation. B3LYP/6–31+G* computed Raman spectrum (in acetonitrile) of (II) *trans*-MCH^+^, (III) *cis*-MCH^+^, (IV) *trans*-MC and (V) *cis*-MC.

The Raman spectrum of solid merocyanine for the region 350–1650 cm^−1^ is shown in Figure 5A. The observed Raman bands are assigned to the stretching and bending vibrations of the pyridinium (py) ring, phenoxyl (ph) ring and to the ethylenic (C=C)_eth_ group joining the two rings. The assignments are based on comparison of the observed vibrations with the computed (B3LYP/6–31+G* and B3LYP/DGDZVP) frequencies for *trans*-MCH^+^ and *cis*-MCH^+^. All the observed Raman vibrations of solid MC along with the computed vibrations are shown in Table 1. Both, *trans*-MCH^+^ and *cis*-MCH^+^ comprise of 36 atoms and thus, contain 102 fundamental modes of vibrations. The conformers belong to the C_1_ point group and all the fundamental vibrations are expected to appear both in Raman and infrared spectra. It is observed from Figure 5A and Table 1 that the Raman spectrum of solid MC exhibits two strong marker bands at 1534 and 1132 cm^−1^ that are assigned to ethylenic (C=C)_eth_ stretching (str) in combination with phenoxyl (ph) ring (CC)_ph_ str and in-plane (ip) (HCC)_eth_ bend and C_26_N_25_ str combined with C_2_C_4_ and C_1_C_3_ str, respectively. Medium intensity Raman bands are observed at 1562, 1292, and 1166 cm^−1^, which are assigned to (C=C)_eth_ str combined with (CC)_ph_ str and ip (HCC)_eth_ bend, ip (HCC)_eth_ bend, ip (HCC)_ph_ bend and pyridinium (py) ip (HCC)_py_ bend and ip (CCC)_py_ bend and ip (HCC)_py_ bend, respectively. Weak bands are observed at 1450, 496, and 435 cm^−1^. Of these vibrations, the mode observed at 1450 cm^−1^ corresponds to the asymmetric (asym) (CC)_ph_ str combined ip (HCC)_eth_ and ip (HCC)_ph_ bend. The 496 and 435 cm^−1^ bands are assigned to ring rotation corresponding to ph and py groups and out-of-plane (oop) (COH)_ph_ ring. A comparison of the solid Raman spectrum with the theoretically computed (B3LYP/6–31+G* and B3LYP/DGDZVP) Raman spectrum of the *trans-* and *cis*-conformers of MCH^+^ is displayed in Supplementary Figure S1. The figure clearly shows the resemblance of the computed Raman spectrum of *trans*-and *cis*-forms of MCH^+^ with the Raman spectrum of solid merocyanine. This indicates that in solid, merocyanine mainly exists as *trans*-MCH^+^ and *cis*-MCH^+^. It is observed from Supplementary Figure S1 that the computed Raman spectrum of *trans*-MCH^+^ and *cis*-MCH^+^ is almost identical for both 6–31+G* and DGDZVP basis sets. Thus, for the sake of brevity, in Figure 5A, the solid Raman spectrum of merocyanine is compared with the B3LYP/6–31+G* computed Raman spectrum of the *trans*- and *cis*-conformers of MCH^+^. The computed Raman spectrum of the *trans*- and *cis*-conformers of the de-protonated form (MC) is also included in Figure 5A for comparison with the solid Raman spectrum of merocyanine. It is clearly evident from the figure that in solid state, both the *trans*- and *cis*-conformers of merocyanine predominantly exists in the protonated (MCH^+^) form.

**TABLE 1:** Assignments of Raman spectrum in solid, acetonitrile and SERRS spectrum of merocyanine along with the B3LYP/6–31+G* computed vibrations (in cm^−1^) of MCH^+^.

Raman		SERRS	Computed vibrations B3LYP/6–31+G*		Assignments
Solid	Solution		*trans*-MCH^+^	*cis*-MCH^+^	
		1612w	1613	1606	ν(CC)_py_,ν(CC)_eth_, δ(HCC)_py_
		1580w	1587	1580	ν(CC)_ph_,ν(CC)_eth_, δ(HCC)_ph_
1562 m	1565 m		1547	1545	ν(CC)_eth_,ν(CC)_ph_, δ(HCC)_eth_
1534s	1538s	1540s	1529	1526	
	1497w		1493	1496	ν(CC)_py_,ν(CN)_py_, δ(HCC)_py_
1450w	1450w	1440w	1416	1412	ν(CC)_ph_ asym,δ(HCC)_eth_, δ(HCC)_ph_
		1397w	1348	1342	ν(CC)_py_ asym,δ(HCC)_py_
1292 m	1290w	1274 m	1298	1305	δ(HCC)_eth_, δ(HCC)_ph_, δ(HCC)_py_
1166 m	1169 m	1174 m	1176	1181	δ(CCC)_py_, δ(HCC)_py_
1132s	1133s 1109 m	1126s	1127	1111	ν (C_26_N_25_), ν (C_2_C_4_), ν (C_1_C_3_), δ(HCC)_py_
		937w	952	950	τ(HCCH)_eth_
		854 m	821	805	C-O str, (ring breathing)_ph_, (ring breathing)_py_
		705w	696	739	(ring distorsion)_py_, CH_2_ rock
		578w	564	610	δ(CCC)_ph_, δ(CCC)_py_, CH_2_ rock
		562w			
496w		466w	468	470	(ring rotation)_ph_, (ring rotation)_py_
435w			422	416	γ(COH)_ph_

Abbreviations useds: strong, m, medium; w, weak; ν, stretching, δ: in-plane bending, τ: torsion, γ: out-of-plane bending, py: pyridinium ring, eth: ethylenic (C=C)_eth_ group, ph: phenoxyl ring.

Since, the resonance Raman (RR) spectrum of merocyanine (10 mM) in acetonitrile solution recorded with 514.5 nm excitation was completely masked by the huge fluorescence background, the Raman spectrum was recorded at 785 nm excitation and the spectrum is shown in Supplementary Figure S2A. The dominance of the acetonitrile peaks is clearly observed from the figure. In order to get meaningful data in solution, the Raman spectrum of acetonitrile (Supplementary Figure S2B) was subtracted from the spectrum of merocyanine in acetonitrile. The subtracted spectrum of merocyanine in acetonitrile for the region, 350–1650 cm^−1^ is also included in Figure 5B for comparison with the solid Raman spectrum. The observed Raman vibrations of merocyanine in solution are also displayed in Table 1. From Figure 5B and Table 1, it is seen that intense marker bands are observed at 1538 and 1133 cm^−1^ that are assigned to (C=C)_eth_ str in combination with (CC)_ph_ str and ip (HCC)_eth_ bend and C_26_N_25_ str combined with C_2_C_4_ and C_1_C_3_ str, respectively. Medium intensity Raman bands are observed at 1565, 1169, and 1109 cm^−1^, which are assigned to (C=C)_eth_ str in combination with (CC)_ph_ str and ip (HCC)_eth_ bend, ip (CCC)_py_ bend along with ip (HCC)_py_ bend and C_26_N_25_ str combined with C_2_C_4_ and C_1_C_3_ str, respectively. Weak Raman bands are observed at 1497, 1450, and 1290 cm^−1^. Of these vibrations, the mode observed at 1497 cm^−1^ is assigned to (CC)_py_ str, (CN)_py_ str and ip (HCC)_py_ bend. The modes at 1450 and 1290 cm^−1^ are assigned to (CC)_ph_ asym str combined with ip (HCC)_eth_ bend and ip (HCC)_ph_ bend and ip (HCC)_eth_ bend, ip (HCC)_ph_ bend and ip (HCC)_py_ bend, respectively. Assessment of the solution Raman spectrum (Figure 5B; Table 1) and comparison with the solid and computed Raman spectrum (in acetonitrile) of the *trans*-MCH^+^ and *cis*-MCH^+^ clearly suggests that majority of the merocyanine peaks in solution are similar to the solid spectrum, which confirms the presence of both the *trans*-MCH^+^ and *cis*-MCH^+^ in solution. In order to verify the existence of the deprotonated form (MC) in solution, the Raman spectrum of merocyanine in acetonitrile solution (Figure 5B) is compared with the computed Raman spectrum of the *trans*-MC (Figure 5BIV) and *cis*-MC (Figure 5BV). The figure clearly shows the abundance of the *trans*- and *cis*-conformers of the protonated form, MCH^+^ in solid and acetonitrile solution and negligible contribution from the deprotonated forms. Overall, a good agreement between the computed Raman spectrum of *trans*-MCH^+^ and *cis*-MCH^+^ with the experimental Raman spectrum in solid and acetonitrile solution is observed.

### Surface-Enhanced Resonance Raman Scattering Spectrum of Merocyanine

**FIGURE 6:**
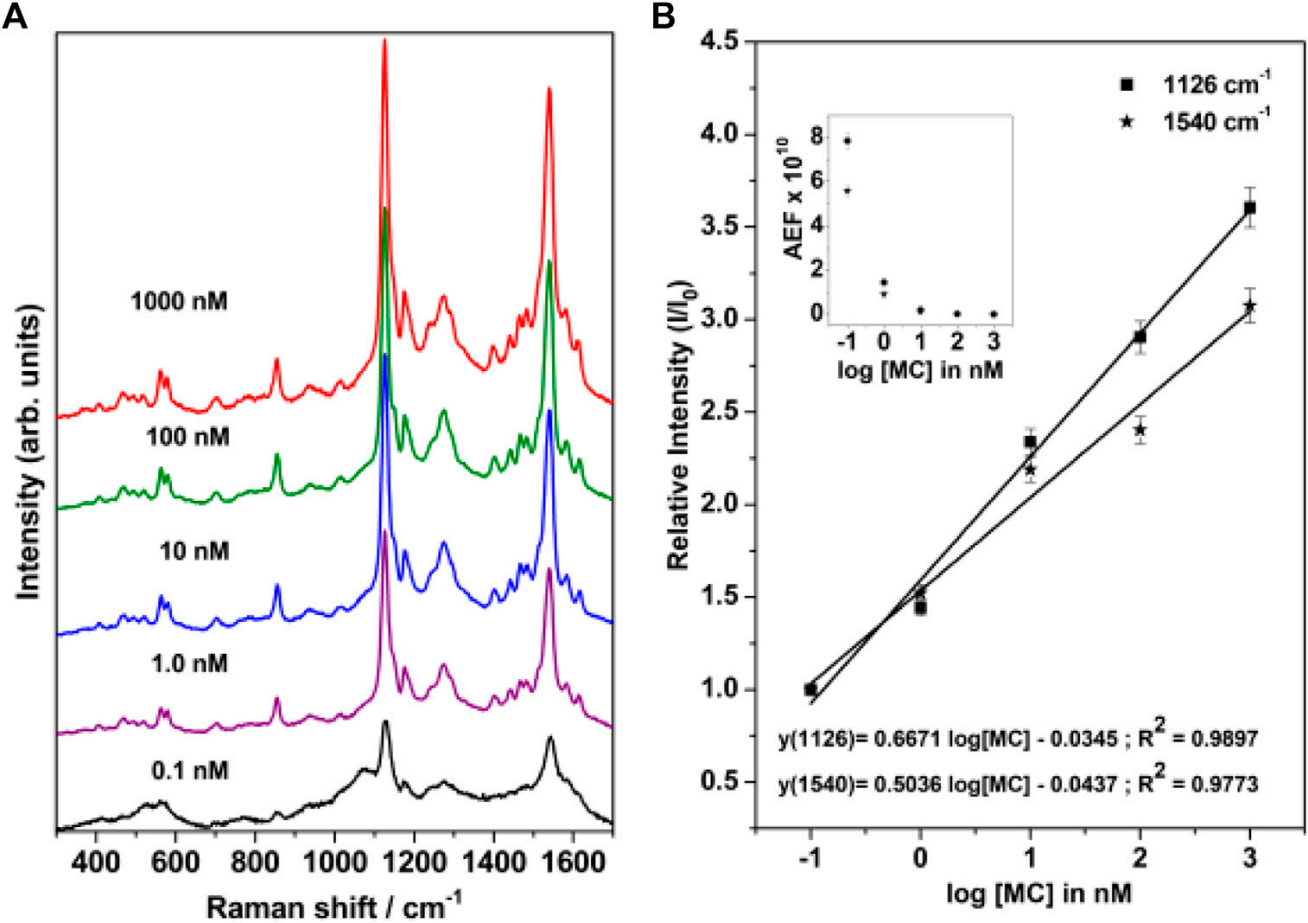
**(A)** Concentration-dependent SERRS spectrum of merocyanine recorded at 514.5 nm excitation. **(B)** Relative SERRS intensity response (I/I_0_) of the 1,126 and 1,540 cm^−1^ bands of merocyanine as logarithmic function of the MC concentration. The inset in Figure 6B shows the analytical enhancement factor (AEF) corresponding to the 1,126 and 1,540 cm^−1^ Raman bands as a function of merocyanine concentration.

The concentration-dependent Surface-Enhanced Resonance Raman Scattering (SERRS) spectrum of merocyanine adsorbed on nanostructured silver-coated films (SCFs) are shown in Figure 6A. The concentration of merocyanine was varied from 0.1 to 1000 nM. The SERRS measurements were performed on the dried surface of the silver films. From Figure 6A, it is evident that modest enhancement of Raman bands are observed at 0.1 nM concentration of merocyanine. Appreciable enhancement in the intensities of the Raman bands are observed at 1, 10, 100, and 1,000 nM concentrations of merocyanine. Maximum enhancement is observed at the merocyanine concentration of 1,000 nM, probably due to monolayer coverage of the analyte on the nanostructured SCFs. At lower concentrations of merocyanine, the SERRS intensity was less, possibly due to the sub-monolayer coverage. Intense marker bands in SERRS spectrum are observed at 1540 and 1126 cm^−1^. Of these vibrations, 1540 cm^−1^ is assigned to the (C=C)_eth_ str combined with (CC)_ph_ str and ip (HCC)_eth_ bend and 1126 cm^−1^ is attributed to the C_26_N_25_ str combined with C_2_C_4_ and C_1_C_3_ str. Medium and weak SERRS bands are observed at 1612 [(CC)_py_ str, (CC)_eth_ str and ip (HCC)_py_ bend], 1580 [(CC)_ph_ str, (CC)_eth_ str and ip (HCC)_ph_ bend], 1440 [(CC)_ph_ str, asym (HCC)_eth_ bend and ip (HCC)_ph_ bend], 1397 [asym (CC)_py_ str, and ip (HCC)_py_ bend], 1274 [ip (HCC)_eth_ bend, ip (HCC)_ph_ bend and ip (HCC)_py_ bend], 1174 [ip (CCC)_py_ bend and ip (HCC)_py_ bend), 937 [(HCCH)_eth_ torsion], 854 [CO str, (ring breathing)_ph_ and (ring breathing)_py_], 705 [(ring distortion)_py_ and CH_2_ rock], 578 [ip (CCC)_ph_ bend, ip (CCC)_py_ bend and CH_2_ rock], 562 cm^−1^ [ip (CCC)_ph_ bend, ip (CCC)_py_ bend and CH_2_ rock] and 466 cm^−1^ [(ring rotation)_ph_ and (ring rotation)_py_)], respectively. All the vibrations observed in SERRS spectrum along with their assignments are tabulated in Table 1. From Figure 6A it is clearly observed that the changes in adsorbate concentration, leads to the overall intensity variation of the Raman bands with absolutely no change in either the band positions or the band widths. This indicates that the adsorbate undergoes no change in the binding characteristics and orientation on the nanostructured SCFs with change in concentration. The relative SERRS intensity response (I/I_0_), normalized for laser power and integration time for the two strong marker peaks observed at 1,126 and 1,540 cm^−1^ as a logarithmic function of the MC concentration is plotted in Figure 6B. The SERRS intensities of 1,126 and 1,540 cm^−1^ bands at MC concentration of 0.1 nM is represented as I_0_ and the intensity for all other concentrations is referred as I. From Figure 6B, it is observed that the relative intensity response (I/I_0_) for both the Raman bands at 1,126 and 1,540 cm^−1^ display linear increase in the logarithmic concentration range of 0.1–1,000 nM. The analytical enhancement factor (AEF) (Le Ru et al., 2007; Pavel et al., 2012; Ben-Jaber et al., 2017) for the 1,126 and 1,540 cm^−1^ bands were calculated from Eq. 1.

$$\;\mathrm{AEF}=\frac{I\left(SERS\right)/C\left(SERS\right)}{I\left(RS\right)/C\left(RS\right)}$$ (1)

where I_(SERS)_ and I_(RS)_ represent the intensity of the average SERS and conventional Raman signal, respectively, and C_(SERS)_ and C_(RS)_ refer to the analyte concentrations in the SERS and Raman measurements, respectively. The plot of AEF as a logarithmic function of MC concentration is shown in the inset of Figure 6B. The AEF for the 1,126 and 1,540 cm^−1^ band are tabulated in Supplementary Table S2. It is observed from Figure 6B and Supplementary Table S2, that the AEF for 1126 cm^−1^ band increases from 2.7 × 10^7^ to 7.9 × 10^10^ as the concentration is lowered from 1000 to 0.1 nM. Similarly, for the 1540 cm^−1^ peak, the AEF varies from 1.8 × 10^7^ to 5.6 × 10^10^ with change in MC concentration from 1000 to 0.1 nM.

**FIGURE 7:**
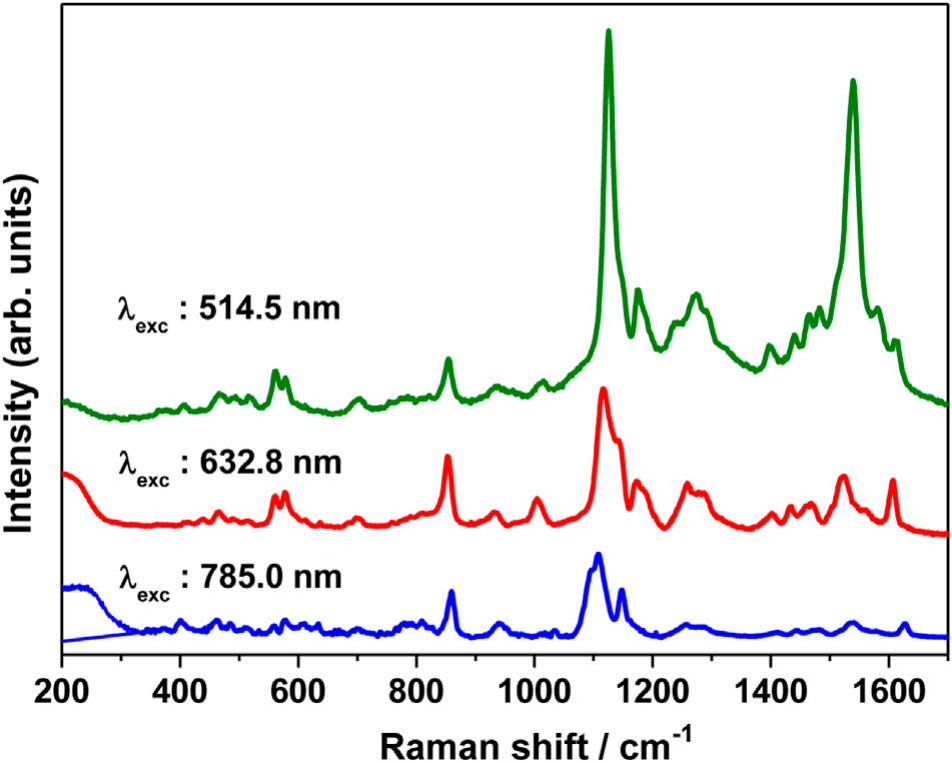
SERS spectrum of merocyanine dye (1 μM) recorded with 785, 632.8, and 785 nm excitation.

The SERS spectrum was also recorded at different excitation wavelengths, viz., 514.5, 632.8, and 785 nm and is shown in Figure 7. The SERS spectrum recorded at 785 nm clearly shows an intense band at 1126 cm^−1^ and moderately intense bands at 1174 and 854 cm^−1^. Weak bands are observed at 1612, 1540, 1274, and 937 cm^−1^. Under pre-resonance conditions, as the excitation wavelength is shifted to 632.8 nm, the SERS bands observed at 562, 578, 854, 1126, 1174, 1274, 1540, and 1612 increases in intensity. Under resonance condition, with the excitation wavelength shifting to 514.5 nm, where the excitation remains in resonance with the intramolecular charge transfer (ICT) transition of merocyanine dye, the SERRS spectrum shows huge enhancement mainly for the 1126 (C_26_N_25_ str combined with C_2_C_4_ and C_1_C_3_ str) and 1540 cm^−1^ [(C=C)_eth_ str combined with (CC)_ph_ str and ip (HCC)_eth_ bend] bands. Thus, from the excitation-dependent SERS spectrum, it is clear that the 1540 cm^−1^ band with weak and moderate intensity at 785 and 632.8 nm excitation displayed huge enhancement under resonance condition (514.5 nm). The 1126 cm^−1^ band that is intense with 785 and 632.8 nm excitation also exhibited huge enhancement under resonance excitation at 514.5 nm. Thus, from the excitation-dependent SERS, it is apparent that in addition to the chemical enhancement due to resonance effect, huge enhancement in intensity for the 1126 and 1540 cm^−1^ bands is observed from the electromagnetic effect; as a result of which not much variation was seen in the spectral shapes of these bands.

**FIGURE 8:**
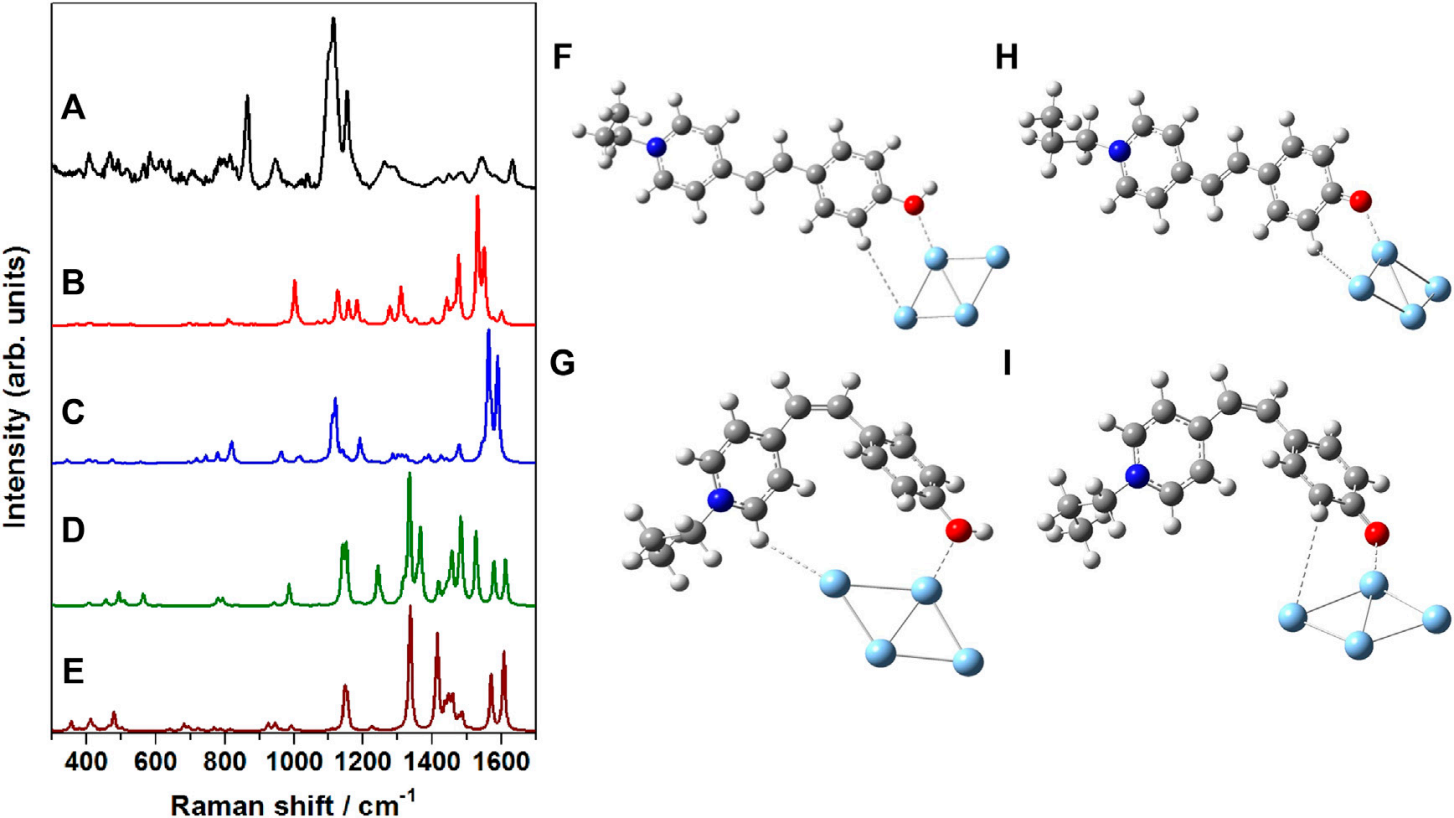
**(A)** SERS spectrum of merocyanine dye (1 μM) recorded at 785 nm excitation, B3LYP/6–31+G* computed Raman spectrum of **(B)** *trans*-MCH^+^-Ag_4_, **(C)** *cis*-MCH^+^-Ag_4_, **(D)** *trans*-MC-Ag_4_ and **(E)** *cis*-MC-Ag_4_. Optimized molecular structures of **(F)** *trans*-MCH^+^-Ag_4_, **(G)** *cis*-MCH^+^-Ag_4_, **(H)** *trans*-MC-Ag_4_ and **(I)** *cis*-MC-Ag_4_.

In order to have a thorough understanding of the binding/adsorption characteristics of the merocyanine dye adsorbed on the nanostructured SCFs, the SERS spectrum recorded at 785 nm excitation (Figure 8A) was compared with the computed Raman spectrum of the Ag_4_ complexes of *trans*-MCH^+^ (Figure 8B), *cis*-MCH^+^ (Figure 8C), *trans*-MC (Figure 8D) and *cis*-MC (Figure 8E). The optimized molecular structures of the *trans*-MCH^+^-Ag_4_, *cis*-MCH^+^-Ag_4_, *trans*-MC-

Ag_4_ and *cis*-MC-Ag_4_ are also included in the Figure 8 (F–I) in order to appreciate the probable binding and orientation of the merocyanine dye on the nanostructured surface of SCFs. On the nanostructured surface, merocyanine may exist in the protonated form (MCH^+^) or it may undergo deprotonation and remain as MC. The prevalence of the *trans-/cis-*conformers of the protonated (MCH^+^) or deprotonated (MC) forms on the nanostructured SCFs surface was confirmed from the observed similarities of the SERS and the computed Raman spectrum of the respective adsorbate (trans-MCH^+^-Ag_4_, cis MCH^+^-Ag_4_, trans MC-Ag_4_ and cis MC-Ag_4_). It is apparent from the figure that the computed vibrational features of *cis*-MCH^+^-Ag_4_ have maximum resemblance with the experimental SERS spectrum albeit differences in the computed Raman intensities. This indicates the predominance of *cis*-MCH^+^ on the surface of nanostructured SCFs although minor contribution from *trans*-MCH^+^ cannot be completely ignored. The figure clearly displays that the computed Raman spectrum of *trans*-MC and *cis*-MC does not have much correlation with the SERS spectrum, which suggests negligible contribution from the deprotonated forms of merocyanine on the nanostructured SCFs.

**FIGURE 9:**
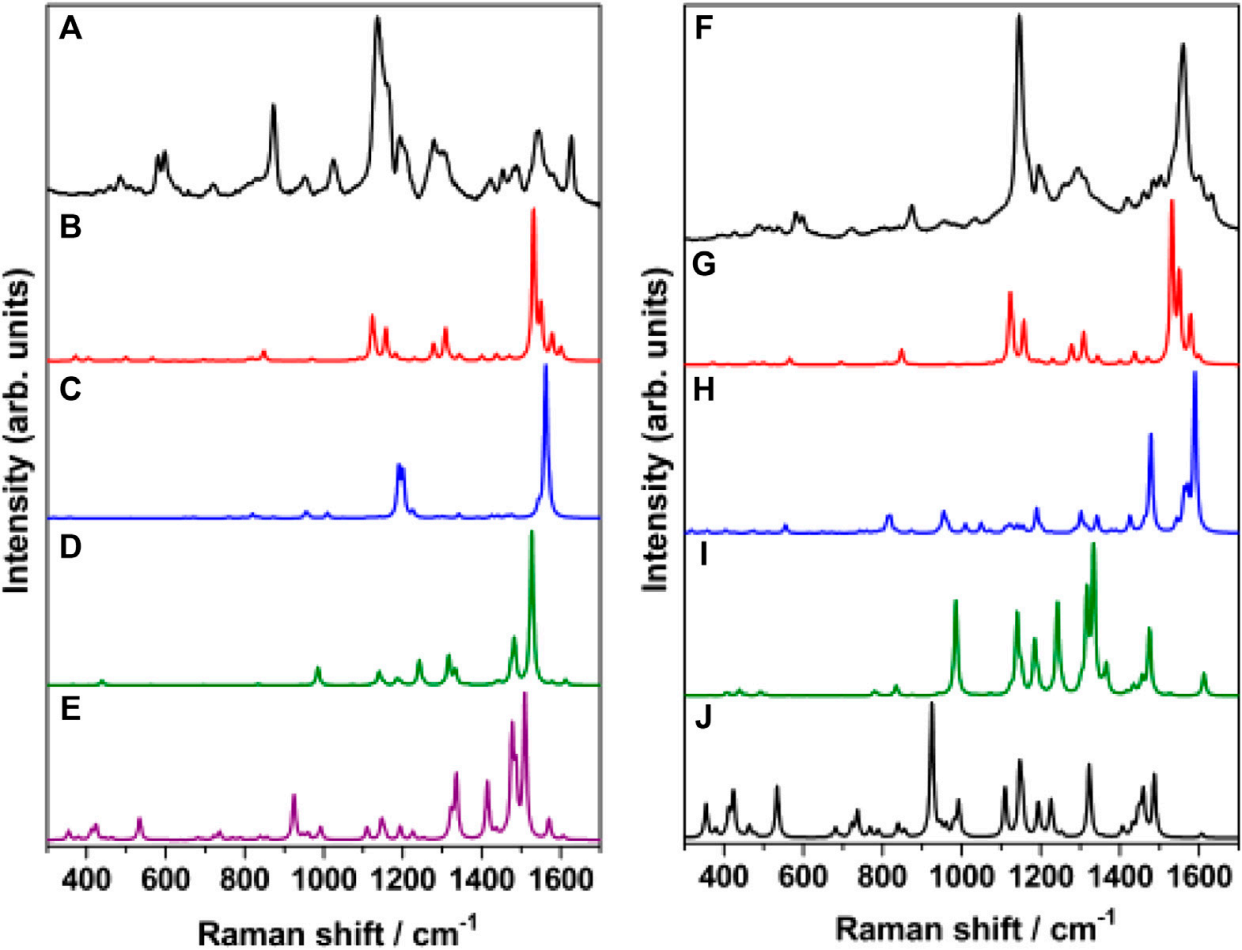
**(A)** SERS spectrum of merocyanine dye (1 μM) recorded at 632.8 nm excitation and B3LYP/6–31+G* computed Raman spectrum (632.8 nm) of **(B)** *trans*-MCH^+^-Ag_4_, **(C)** *cis*-MCH^+^-Ag_4_, **(D)** *trans*-MC-Ag_4_ and **(E)** *cis*-MC-Ag_4_. **(F)** SERRS spectrum of merocyanine dye (1 μM) recorded at 514.5 nm excitation and B3LYP/6–31+G* computed Raman spectrum (514.5 nm) of **(G)** *trans*-MCH^+^-Ag_4_, **(H)** *cis*-MCH^+^-Ag_4_, **(I)** *trans*-MC-Ag_4_ and **(J)** *cis*-MC-Ag_4_.

In order to identify the sources of intensity enhancement in SERS (Jensen et al., 2008), viz., “long range” (electromagnetic) or “short range” (chemical, resonance with charge-transfer states), the Raman spectrum of *trans*-MCH^+^-Ag_4_, *cis*-MCH^+^-Ag_4_, *trans*-MC-Ag_4_ and *cis*-MC-Ag_4_ was computed under preresonance conditions with respect to molecular excitation energies. The computed Raman spectrum for all the four Ag_4_ complexes with 632.8 and 514.5 nm excitation was compared with the SERS (Figure 9A) and SERRS (Figure 9B) spectrum measured at 632.8 and 514.5 nm, respectively. From Figure 9A, it is evident that the computed Raman spectrum of *trans*-MCH^+^-Ag_4_ under preresonace excitation of 632.8 nm shows reasonable agreement with the experimental SERS spectrum recorded at 632.8 nm, despite differences in intensities. The computed Raman spectrum in pre-resonance condition with excitation at 514.5 nm (Figure 9B) when compared with the SERRS spectrum recorded at 514.5 nm clearly confirm the dominance of the *trans*-MCH^+^-Ag_4_ conformer on the nanostructured SCFs. Although, there are apparent differences in the computed intensities, the Raman activity of *trans*-MCH^+^-Ag_4_ conformer shows good agreement with the SERRS spectrum. In all the cases the computed Raman intensities (activities) differ from the actual measured intensities possibly due to our simplistic “static” or Placzek’s approach (Walter and Moseler, 2020) for computing the Raman activity. Moreover, it is apparent from Figure 9 that the intensity enhancement in the SERS and SERRS spectrum has dominant contribution from the electromagnetic effect, which results in amplification of the Raman intensities without affecting the spectral shape of the Raman bands.

The observed results can be summarized as follows: 1) the prominent marker bands of merocyanine at 1538 (ethylenic C=C stretch) and 1133 cm^−1^ (pyridinium C-N stretch) in acetonitrile solution is shifted to 1540 and 1126 cm^−1^ in the SERRS spectrum. The appreciable shift of 7 cm^−1^ for the pyridinium C-N stretching vibration is also evident from the observed change in the bond distance of C_26_N_25_ from 1.489 Å (*trans*-MCH^+^/*cis*-MCH^+^) to 1.5 Å (*cis*-MCH^+^-Ag_4_/*trans*-MCH^+^-Ag_4_). 2) The marker bands of merocyanine seen at 1540 and 1126 cm^−1^ are very intense in the SERRS spectrum, although the 1540 cm^−1^ band is much weaker in the SERS spectrum measured at 632.8 and 785 nm. These results suggest that while the 1126 cm^−1^ band is enhanced mainly due to the electromagnetic effect, the 1540 cm^−1^ peak displays resonance enhancement in addition to the electromagnetic effect. 3) The excitation-dependent SERS spectrum thus, infers that in addition to the major contribution from the electromagnetic enhancement, chemical (resonance) effect also leads to amplification of the 1540 cm^−1^ band. 4) The comparative study of the excitation-dependent SERS study and the computed Raman activity under static and in preresonance excitation conditions confirm the predominance of the *trans*-MCH^+^-Ag_4_ form bound to the nanostructured surface via the phenoxyl ring O atom with minor contribution from the *cis*-MCH^+^-Ag_4_ conformer.

## Conclusion

In this article, a comprehensive structural and vibrational analysis of merocyanine is reported in solid, acetonitrile solution and adsorbed on the nanostructured surface of silver-coated films (SCFs) using Raman scattering in combination with DFT studies. The Raman spectrum of solid merocyanine in combination with DFT calculations inferred the existence of the *trans*- and *cis*-conformers of the protonated form of merocyanine (MCH^+^) in the solid state. The Raman study of merocyanine in acetonitrile solution indicated major contribution from the *trans*- and *cis*-conformers of MCH^+^ remaining in equilibrium, although the computed absorption spectrum in acetonitrile indicated the prevalence of the cis-MC conformer in acetonitrile. The presence of the protonated forms of merocyanine in solid and acetonitrile solution confirm the predominance of benzenoid structure. The prominent marker bands, observed in the Raman spectrum of merocyanine in acetonitrile at 1538 (ethylenic C=C stretch) and 1133 cm^−1^ (pyridinium C-N stretch) were shifted to 1540 and 1126 cm^−1^ on the nanostructured SCFs. The appearance of the marker bands as well as the band shifts in SERRS is associated with the selective binding of the *trans*-MCH^+^ conformer on the nanostructured metal surface that assumes benzenoid structure and is exclusively bound via the active anchoring site, viz., the phenoxyl group O atom. The concentration-dependent SERRS spectrum of merocyanine functionalized SCFs showed maximum enhancement at 1 μM concentration for all observed vibrations indicating monolayer coverage of the adsorbate. The SERRS study also revealed sub-nanomolar (0.1 nM) sensing of merocyanine using nanostructured SCFs with the analytical enhancement factor (AEF) of ∼ 10^10^ for the 1126 cm^−1^ and 1540 cm^−1^ Raman bands for MC concentration of 0.1 nM. The excitation-dependent SERS study infers that in addition to the major contribution from the electromagnetic enhancement, chemical (resonance) effect leads to the amplification of the 1540 cm^−1^ band. The observed conformational surface selectivity of the *trans*-isomer of protonated merocyanine using nanostructured surfaces can be further exploited for energy efficient and economical separation of geometrical isomers.

## Data Availability

The original contributions presented in the study are included in the article/Supplementary Material, further inquiries can be directed to the corresponding author.
