# Supplementary material for: Conformational Selectivity of Merocyanine on Nanostructured Silver Films: Surface Enhanced Resonance Raman Scattering (SERRS) and Density Functional Theoretical (DFT) Study
Source: Front Chem. 2022 Jun 13;10:902585. doi: 10.3389/fchem.2022.902585 (PMC9234333; doi:10.3389/fchem.2022.902585)
Supplement: Supplementary file 1 [file DataSheet1.PDF]

Conformational selectivity of merocyanine on nanostructured silver films: Surface enhanced resonance Raman scattering (SERRS) and Density Functional Theoretical (DFT) Study

Abhishek Das[a], Ridhima Chadha[a], Amaresh Mishra[b] and Nandita Maiti*[a,c]

[a]Radiation & Photochemistry Division, Bhabha Atomic Research Centre, Mumbai-400085, India

[b]Department of Chemistry, Sambalpur University, Jyoti Vihar, Sambalpur 768 019, Orissa

[c]Homi Bhabha National Institute, Anushaktinagar, Mumbai-400094, India

Supplementary Material

Table S1: Optimized (B3LYP/6-31+G*; B3LYP/LANL2DZ for Ag) parameters of *trans*-MCH^+, *cis*-MCH^+, *trans*-MC, *cis*-MC and their Ag_4 complexes.

Bond Distances, Bond Angles & Dihedral Angles	*trans*-MCH^+	*trans*-MCH^+-Ag_4	*cis*-MCH^+	*cis*-MCH^+-Ag_4	*trans*-MC	*trans*-MC-Ag_4	*cis*-MC	*cis*-MC-Ag_4
C_1C_2	1.368	1.376	1.368	1.367	1.409	1.407	1.418	1.411
C_1C_3	1.441	1.454	1.453	1.476	1.394	1.414	1.395	1.419
C_2C_4	1.436	1.445	1.447	1.469	1.395	1.411	1.397	1.421
C_3C_5	1.414	1.422	1.413	1.416	1.444	1.444	1.446	1.445
C_3C_7	1.418	1.426	1.415	1.419	1.445	1.445	1.445	1.443
C_4C_6	1.423	1.432	1.421	1.427	1.445	1.449	1.446	1.448
C_4C_8	1.422	1.434	1.419	1.422	1.446	1.451	1.445	1.447
C_5C_{11}	1.387	1.399	1.389	1.406	1.361	1.378	1.361	1.379
C_7C_9	1.381	1.395	1.384	1.398	1.360	1.376	1.361	1.377
C_6C_{12}	1.373	1.383	1.375	1.386	1.361	1.372	1.361	1.375
C_8C_{10}	1.373	1.382	1.376	1.389	1.361	1.374	1.362	1.375
C_9C_{13}	1.409	1.409	1.407	1.405	1.469	1.455	1.469	1.454
$C_{11}C_{13}$	1.404	1.408	1.403	1.404	1.466	1.452	1.466	1.452
$C_{10}N_{25}$	1.361	1.377	1.360	1.374	1.376	1.387	1.376	1.385
$C_{12}N_{25}$	1.362	1.378	1.359	1.373	1.379	1.389	1.377	1.385
$C_{26}N_{25}$	1.488	1.499	1.489	1.500	1.469	1.485	1.470	1.488
$C_{13}O_{14}$	1.350	1.403	1.353	1.414	1.244	1.299	1.244	1.300
$O_{14}Ag$		2.410		2.418		2.232		2.244
$C_1C_2C_4$	125.5	125.5	131.5	128.5	125.7	125.6	130.9	130.8
$C_2C_1C_3$	127.7	127.1	132.7	127.9	128.1	127.6	132.3	131.9
$C_1C_3C_5$	118.6	118.6	118.3	120.3	119.4	119.3	119.1	119.1
$C_1C_3C_7$	123.7	123.4	123.9	121.2	123.9	123.7	124.4	124.0
$C_2C_4C_6$	119.6	119.8	118.8	119.8	120.7	120.5	119.9	119.6
$C_2C_4C_8$	125.1	124.6	125.5	123.8	125.6	125.1	126.2	125.9
$H_{15}C_1C_2H_{16}$	179.9	-179.4	-8.2	-6.9	179.9	-179.9	-21.1	-16.8
$C_3C_1C_2C_4$	-179.9	-179.4	-14.4	-11.2	179.9	179.9	-31.1	-24.8
$C_2C_1C_3C_5$	179.8	-178.2	155.8	142.1	179.9	-179.8	169.6	164.9
$C_2C_1C_3C_7$	-0.1	2.3	-28.8	-39.4	-0.0	0.1	-14.1	-17.9
$C_1C_2C_4C_6$	179.8	-179.4	166.5	154.0	179.8	179.9	177.4	174.5
$C_1C_2C_4C_8$	0.1	0.9	-19.0	-30.0	0.2	0.2	-7.7	-10.7

Table S2: The analytical enhancement factor (AEF) calculated for the intense SERRS bands, observed at 1126 and 1540 cm^{-1}.

MC concentration	AEF (1126)	AEF (1540)
1000 nM	2.7×10^{7}	1.8×10^{7}
100 nM	2.1×10^{8}	1.3×10^{8}
10 nM	1.9×10^{9}	1.2×10^{9}
1 nM	1.4×10^{10}	8.9×10^{9}
0.1 nM	7.9×10^{10}	5.6×10^{10}

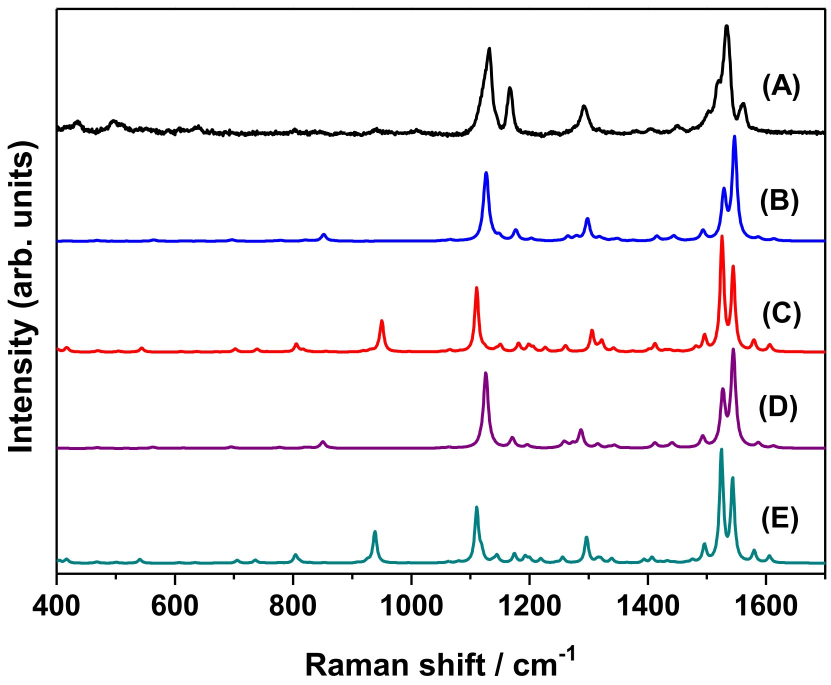

Figure S1. Raman spectrum of merocyanine in (A) solid recorded at 785 nm excitation. B3LYP/6-31+G* computed Raman spectrum of (B) *trans*-MCH^+ (C) *cis*-MCH^+, B3LYP/DGDZVP computed Raman spectrum of (D) *trans*-MCH^+ and (E) *cis*-MCH^+.

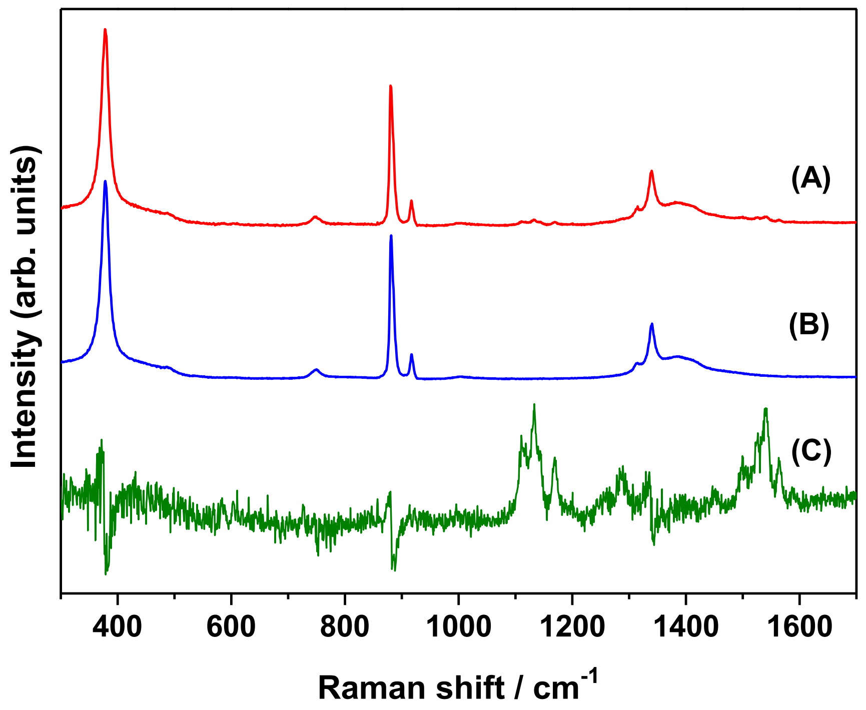

Figure S2. Raman spectrum of (A) merocyanine in acetonitrile, (B) acetonitrile (C) subtracted spectrum of merocyanine in solution [(A)-(B)]. The Raman spectrum was recorded at 785 nm excitation.
